# Albumin to prealbumin ratio in peritoneal dialysis patients: Clinical implication and outcome prediction

**DOI:** 10.1371/journal.pone.0276159

**Published:** 2022-11-08

**Authors:** Yun-Ting Huang, Ming-Yan Jiang, Jyh-Chang Hwang

**Affiliations:** 1 Division of Nephrology, Chi Mei Medical Center, Tainan, Taiwan; 2 Department of Pharmacy, Chia Nan University of Pharmacy & Science, Tainan, Taiwan; Obafemi Awolowo University, NIGERIA

## Abstract

**Background:**

Serum prealbumin level is slightly higher, whereas albumin is lower in peritoneal dialysis (PD) than hemodialysis (HD) patients. It is unknown whether albumin to prealbumin ratio (APR) is associated with mortality risk among PD patients. This study aimed to evaluate the clinical implications of APR and its prediction value on long-term outcomes of PD patients.

**Methods:**

The study population were prevalent PD patients at a tertiary hospital. Based on APR, a total of 220 PD patients were divided into 3 groups: group 1: top tertile, median APR: 121.1; IQR:109.5–131.9 (n = 73, male: 37%; age: 59±13); group 2: middle tertile, median APR: 97.1; IQR 93.5–100.0 (n = 73, male:37%; age: 54±14), and group3: bottom tertile, median APR: 81.3; IQR:76.8–85.0 (n = 74, male:38%; 54±11). Patients were followed up for a maximum of 5 years. Outcome of interest was all-cause mortality.

**Results:**

Group 1 was characterized by older age, higher prevalence of diabetes, lower nPCR, higher Davies score and hs-CRP level. APR positively correlated to hs-CRP (*β* = 0.149, p = 0.045), but negatively correlated to nPCR (*β* = -0.161, p = 0.034). Hyperprealbuminemia, accounting for 0%, 23.3%, and 82.4% in groups 1,2, and 3, was associated with a lower risk for mortality (HR:0.41, 95%CI = 0.23–0.73). The cumulative survival is significantly lower in group 1 than the other two groups. By multivariable Cox regression, APR (HR:1.02; 95%CI:1.01–1.03) was found to be an independent predictor of long-term mortality.

**Conclusion:**

PD patients with high APR are characterized by having more comorbidities and marked malnutrition-inflammation status, and are associated with long-term mortality, whereas hyperprealbuminemia and lower APR are favorable prognostic factors.

## Introduction

Serum albumin level correlates to nutrition and inflammation status and is a well-known predictor of long-term outcomes among individuals with chronic kidney disease (CKD) [1]. Both malnutrition and inflammation reduce serum albumin level by decreasing synthesis and accelerating degradation [2]. Studies have shown that hypoalbuminemia is associated with long-term adverse health consequences among end stage renal disease (ESRD) patients [3].

Prealbumin, also called transthyretin, is a transport protein of thyroid hormone. Similar to albumin, prealbumin is a negative acute-phase reactant, and low level may indicate either inadequate nutrition or excessive inflammatory load [4]. Compared with albumin, prealbumin has a shorter half-life and is synthesized more rapidly in the liver, and thus its catabolic rate is more predictable [5]. Studies have demonstrated that low prealbumin level was associated with increased risk of hospitalization and death both in hemodialysis (HD) [6] and peritoneal dialysis (PD) patients [7]. In addition, declining trajectory of serum prealbumin had been shown to independently predict long-term mortality among individuals receiving HD [8].

Both serum albumin and prealbumin are negative acute-phase reactants and indicators of nutritional status in dialysis patients and are positively correlated with each other [9]. However, a previous study showed that serum prealbumin level among PD patients is higher by about 6mg/dL in average while serum albumin is approximately 0.3 g/dL lower when compared with HD patients, perhaps due to peritoneal albumin loss with stimulated hepatic synthesis of prealbumin [10]. The clinical significance of higher prealbumin but lower albumin levels among PD patients remains unknown. Whether the ratio of serum albumin to prealbumin (APR) is associated with long-term prognosis in PD patients has never been investigated. Our study purpose is to explore the implication of higher prealbumin as opposed to lower albumin and examine the association between APR and long-term mortality among ESRD patients undergoing PD.

## Materials and methods

The study population consist of 221 prevalent ESRD patients undergoing PD for at least 3 months at a tertiary hospital. Eighty-five percent of patients were dialyzed with 4 or 5 sessions of Dianeal PD solution per day through Twin-Bag system, and 15% used automatic peritoneal dialysis (Baxter Healthcare Corporation, Singapore) for daily renal replacement therapy. Complete blood count and serum biochemistry tests including albumin were checked monthly (Abbott Laboratories, Abbott Park, IL), while serum prealbumin data (Abbott Laboratories, Abbott Park, IL) was collected bimonthly during regular clinic visits. Patients who had at least 5 test results of albumin and 2 results of prealbumin from January 2016 to June 2016 were included. Data obtained during hospitalization stay or at emergency service were excluded. After excluding 1 individual who had insufficient albumin/prealbumin test results, a total of 220 patients were eligible to our analysis. The etiologies of renal failure among the 220 patients were chronic glomerulonephritis in 48% of patients, diabetic nephropathy in 26%, chronic tubulointerstitial nephritis in 11%, hypertension in 11%, and others in 4%, respectively.

APR was calculated as mean albumin divided by mean prealbumin in unified unit, i.e., mg/dL. We then divided the study population into 3 groups by tertiles based on the APR: group 1: top tertile (n = 73) with median APR of 121.1 [interquartile range (IQR):109.5–131.9]; group 2: middle tertile (n = 73) with median APR of 97.1 (IQR 93.5–100.0); group3: bottom tertile (n = 74) with median APR of 81.3 (IQR:76.8–85.0).

Data at baseline including demographic information, PD treatment vintage, urine output, daily ultrafiltration, normalized protein catabolism rate (nPCR, Randerson formula [11]), Kt/V, renal creatinine clearance (renal CCr, calculated by collecting 24hr urine volume times urine Cr divided by serum Cr levels), mean arterial pressures (MAP), body mass index (BMI), biochemistry panel, as well as comorbid conditions, were ascertained from electronic medical records. Comorbidities were weighed by Davies comorbidity score [12] and were recorded as diabetes (yes or no) and Davies score with extraction of diabetes (ordinal variable). Outcome of interest was all-cause mortality. Patients with kidney transplantation, transferal to other centers, or shifting to hemodialysis modality were recorded as censor. Patients were followed up until censored or June 30, 2021. All patients’ records/information were anonymized and de-identified prior to analysis. The current study was approved by the Ethics Committee of Chi Mei Medical Center (no. 11102–008) and was conducted in accordance with the guiding principles for human experimentation of the Helsinki Declaration.

### Statistical analysis

**Fig 2:**
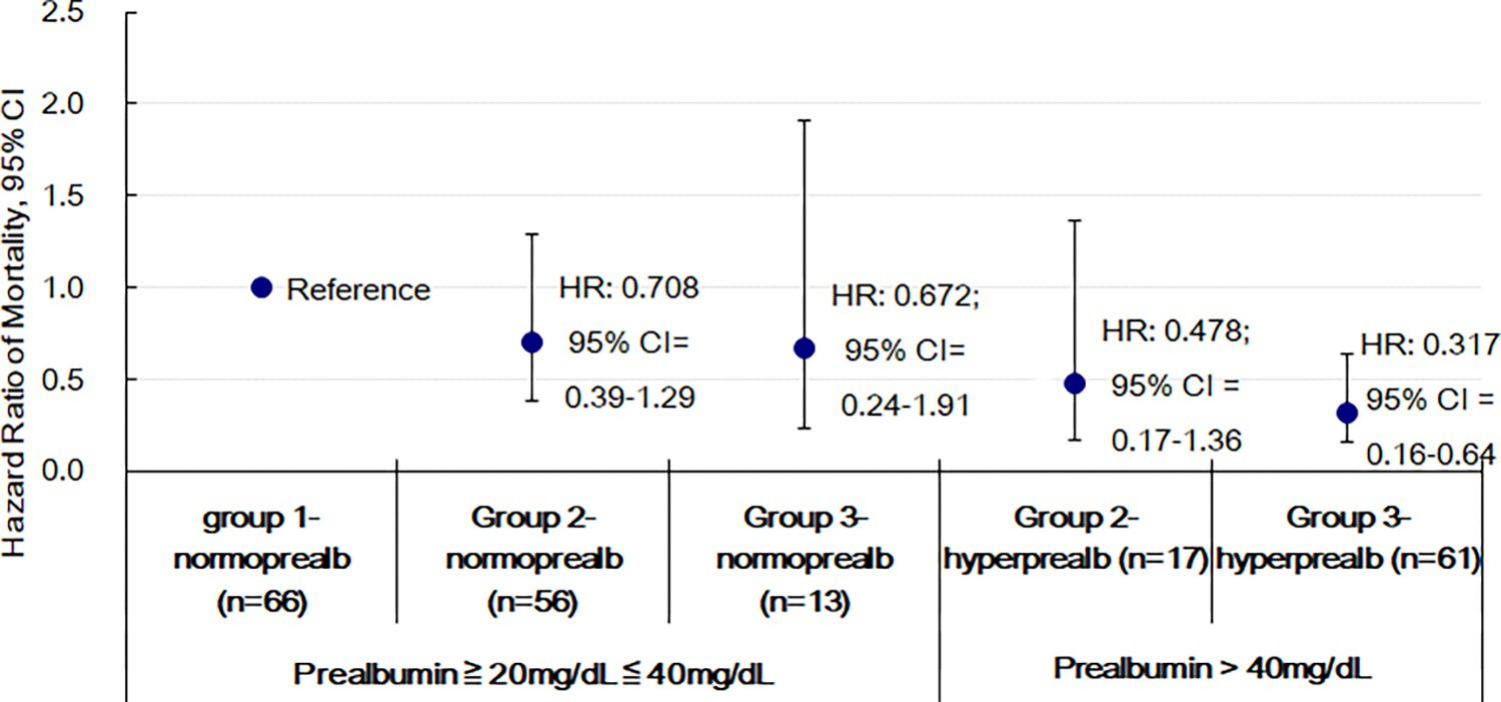
Hazard ratios of mortality stratified by prealbuminemia level. As opposed to group 1 with normoprealbuminemia (reference), groups 2 and 3 lost their survival benefit in normoprealbuminemia panel. Whereas in hyperprealbuminemia panel, individuals in group 3 had significantly lower mortality risk than reference group, but group 2 with only 17 in patient number, did not show any difference compared to group 1.

Pearson correlation was conducted to evaluate the association between serum albumin and prealbumin levels. Chi-square test, one way analysis of variance (ANOVA) test or Kruskal-Wallis test were used for comparisons for the respective categorical and continuous variables among three groups in Table 1. Clinical factors related to APR were evaluated by simple and multiple linear regression analyses in Table 2. Age at the start of study, sex, PD vintage, nPCR, hs-CRP level and Davies score with extraction of DM were selected into model 1 regression analysis. In model 2, we further included DM in the multivariable regression because of significantly higher prevalence of DM in group 1. In Table 3, we compared the characteristics between individuals with hyperprealbuminemia (prealbumin > 40mg/dL) and normoprealbuminemia (20-40mg/dL) by unpaired t test or Mann–Whitney U test to highlight the specific features in those with prealbumin more than 40mg/dL. Actuarial survival curves of the three groups were determined by the Kaplan-Meier methods with Log rank tests. In Fig 2, we performed a subgroup analysis to investigate the effect of prealbumin on long-term survival, in which only patients with prealbumin level of ≥ 20 mg/dL were included. The three APR groups were further stratified as normo- or hyperprealbuminemia to compare the mortality risk by Cox regression analysis; group1 with normoprealbuminemia was served as the reference group.

**Table 1:** Baseline characteristic of the three groups of patients.

	Group1	Group 2	Group 3	
	n = 73	n = 73	n = 74	*p*
**Demographic factors**				
Diabetes mellitus, n (%)	28 (38.4)	17 (23.3)	12 (16.2)	0.008
Male, n (%)	27 (37)	27 (37)	28 (38)	0.99
Age at study, years	59±13	54±14	54±11	0.05
PD vintage, months	56.5±37.1	60.4±41.1	56.6±40.0	0.54
Urine output>750 mL/day, n (%)	14 (19.2)	9 (12.3)	26 (35.1)	0.003
Ultrafiltration, mL/day	1036±488	1055±492	922±511	0.23
nPCR*, g/kg/day	1.00±0.22	1.12±0.27	1.08±0.25	0.01
Kt/V	1.93±0.32	1.99±0.34	1.90±0.21	0.21
Renal CCr, ml/min	8.19±24.26	3.31±7.51	6.86±13.65	0.26
Mean arterial pressure, mmHg	105.2±19.2	107.6±17.3	106.4±17.9	0.72
BMI, kg/m^2^	22.8±1.8	20.0±3.6	21.3±2.9	0.09
**Clinical comorbidity, n (%)**				
Coronary artery disease	16 (21.9)	12 (16.4)	11 (14.9)	
Congestive heart failure	8 (11.0)	7 (9.6)	4 (5.4)	
Peripheral vascular disease	6 (8.2)	2 (2.7)	3 (4.1)	
Stroke	12(16.4)	6 (8.2)	3 (4.1)	
Neoplasm	7 (9.6)	10 (13.7)	15 (20.3)	
Chronic lung disease	3 (4.1)	1 (1.4)	0 (0.0)	
Liver cirrhosis and/or hepatoma	3 (4.1)	5 (6.8)	2 (2.7)	
Davies comorbidity score, n (%)				
0	23 (32)	40 (55)	39 (53)	
1	26 (36)	15 (21)	24 (32)	
2	16 (22)	11 (15)	8 (11)	
≧ 3	8 (11)	7 (10)	3 (4)	
**Laboratory data**				
hs-CRP, mg/L, median	5.8	3.0	2.7	<0.001
(1st -3rd quartile ranges)	(2.4–13.2)	(1.2–8.6)	(0.9–6.6)	
Prealbumin, mg/dL	28.8±6.9	38.4±5.4	46.5±6.3	<0.001
(ranges)	(10–43)	(26–52)	(30–60)	
< 20mg/dL, n (%)	7 (9.6)	0 (0)	0 (0)	
20-40mg/dL, n (%)	66 (90.4)	56 (76.7)	13 (17.6)	
> 40mg/dL, %	0 (0)	17 (23.3)	61 (82.4)	
Albumin, g/dL	3.49±0.48	3.63±0.38	3.67±0.37	0.03
(ranges)	2.3–4.7	2.8–4.5	2.7–4.3	
Albumin ≧ 3.5g/dL, n (%)	43 (58.9)	50 (64.5)	55 (74.3)	
Ratio of albumin to prealbumin, median, (1st -3rd quartile ranges)	121.1 109.5–131.9	97.1 93.5–100.0	81.3 76.8–85.0	<0.001
Sodium, mmol/dL	136.3±3.7	135.9±3.4	136.6±4.8	0.52
Potassium, mmol/dL	3.9±0.7	4.1±0.7	4.2±0.7	0.027
Phosphate, mg/dL	5.7±1.5	5.6±1.5	5.8±1.5	0.86
Cholesterol, mg/dL	194±47	197±42	210±48	0.1
BUN, mg/dL	66.9±22.0	75.2±21.8	77.6±22.3	0.01
Creatinine, mg/dL	10.8±3.4	12.1±3.2	12.1±3.4	0.024
Uric acid, mg/dL	6.9±1.4	7.0±1.2	6.7±1.3	0.52
Hemoglobin, g/dL	9.7±1.4	9.7±1.5	9.6±1.6	0.6

Abbreviations: PD: Peritoneal dialysis, nPCR: Normalized protein catabolism rate, CCr: Creatinine clearance, BMI: Body mass index, hs-CRP: High sensitivity C-reactive protein, BUN: Blood urea nitrogen

*: Randerson formula.

**Table 2:** Factors correlated to serum albumin to prealbumin ratio by simple and multiple linear regression analyses.

	Simple Regression		Multiple regression			
			model 1		model 2	
	β	*p*	β	*p*	β	*p*
Age, year	0.147	0.029	0.114	0.137	0.102	0.177
Sex, male = 1	-0.007	0.915	0.042	0.579	0.014	0.857
PD vintage, month	0.036	0.596	0.024	0.751	0.062	0.415
nPCR, g/kg/day	-0.193	0.010	-0.185	0.015	-0.154	0.044
hs-CRP, mg/L	0.225	0.001	0.157	0.037	0.041	0.595
Davies score—DM	0.121	0.073	0.07	0.358	0.14	0.06
DM, yes = 1	0.206	0.002			0.175	0.031

Abbreviations PD: Peritoneal dialysis, nPCR: Normalized protein catabolism rate, hs-CRP: High sensitivity C reactive protein, DM: Diabetes mellitus.

**Table 3:** Difference between hyper-prealbuminemia (>40mg/dL) and normo-prealbuminemia (between 20mg/dL to 40mg/dL).

	Prealbumin >40mg/dL	Prealbumin ≧20mg/dL≦40mg/dL	*p*
	n = 78	n = 135	
Demographic factors			
Diabetes mellitus, n (%)	11 (14)	40 (30)	0.007*
Male, n (%)	30 (38)	51 (38)	0.52
Age at study, years	52±11	57±14	0.005
PD vintage, months	56.5±42.4	56.2±39.3	0.96
nPCR*, g/kg/day	1.12±0.23	1.06±0.26	0.12
Renal CCr, ml/min	6.3±13.2	6.07±18.3	0.92
Mean arterial pressure, mmHg	106.8±15.9	106.8±18.7	0.99
BMI,	20.6±3.0	21.5±3.11	0.44
Davies comorbidity score, n (%)			
0	43 (55.1)	59 (43.7)	
1	26 (33.3)	38 (28.1)	
2	4 (6.4)	24 (17.8)	
> = 3	0 (0.0)	14 (10.4)	
Death rate, n (%)	15 (19.2)	50 (37)	0.005
**Laboratory data**			
hs-CRP, mg/L, median	3.3	5.0	0.017¶
(1st -3rd quartile ranges)	1.11–7.90	2.00–12.7	
Prealbumin, mg/dL	45.7±4.2	32.8±4.9	<0.001
Albumin, g/dL	3.77±0.30	3.46±0.35	<0.001
Ratio of albumin to pre-albumin	82.9±8.1	107.5±17.5	<0.001

Abbreviations PD: Peritoneal dialysis, nPCR: Normalized protein catabolism rate, CCr: Creatinine clearance, BMI: Body mass index, hs-CRP: High sensitivity C reactive protein

*: Randerson formula.

*: Unpaired t test

¶: Mann–Whitney U test.

In Table 4, we conducted Cox regression analysis to examine the difference in mortality risk between groups with adjustment of potential confounders including age, sex, and PD vintage in the model 1. In model 2, we further adjusted for DM to eliminate the potential confounding effect. In Table 5, we constructed a Cox regression model to explore potential predictors of long-term mortality among PD patients, with variables including APR as continuous variable, age at the start of study, DM, Davies score with extraction of DM, MAP, and PD vintage. To evaluate the predictive value of the three parameters, i.e., prealbumin, albumin, and APR, we plot the receiver operating characteristic (ROC) curves and calculate the areas under curve (AUC). AUC of APR was replaced by 1/APR to keep the curve in same direction. The data were expressed as mean ± standard deviation or median (interquartile range). A p-value of less than 0.05 was considered statistically significant. Computations were performed with the SPSS 22.0 package for Windows (SPSS, IBM® SPSS® software).

**Table 4:** Long-term mortality risk among the three groups by Cox regression analysis.

	Model 1				Model 2			
		95% C.I. for HR				95% C.I. for HR		
	HR	Lower	Upper	p-values	HR	Lower	Upper	p-values
Group 1	1				1			
Group 2	0.576	0.336	0.989	0.046	0.660	0.382	1.139	0.136
Group 3	0.379	0.205	0.698	0.002	0.451	0.243	0.838	0.012

Adjusted variables: In model 1: Age, sex, peritoneal dialysis vintage, in model 2: Age, sex, peritoneal dialysis vintage, diabetes mellitus.

## Results

### 1. High albumin to prealbumin ratio (APR) is mainly caused by relatively lower prealbumin level

Among overall PD population, we observed a positive correlation between the serum albumin and prealbumin levels (r = 0.58, p<0.001). After unifying the unit of both parameters into mg/dL, our results showed that the APR ranged from 64.5 to 240.0, with a right skewed distribution. Among individuals with high APR (group 1), we found that 7 patients (9.6%) had hypoprealbuminemia (< 20mg/dL) and 66 patients (90.4%) had normoprealbuminemia, while none had prealbumin level higher than 40mg/dL (Table 1). In addition, more than 80% of individuals in group 3 had prealbumin level of higher than 40mg/dL, and none of patients in group 2 or group 3 had prealbumin level of less than 20 mg/dL. On the other hand, we observed that more than 40% of individuals in group 1 had hypoalbuminemia (albumin < 3.5g/dL), while only one-third and one-fourth in groups 2 and 3, respectively, had albumin lower than 3.5g/dL.

### 2. High serum albumin to prealbumin ratio (APR) is associated with malnutrition, comorbidities, and inflammation

The mean age of the overall population at the start of study was 56±13 years (range 23–92 years) and nearly 40% were males. When categorized by tertiles, our results showed that group 1 tended to be older, higher in prevalence of DM and other comorbidities, and had lower nPCR and higher hs-CRP level (Table 1). In addition, group 1 had lower serum albumin, prealbumin, BUN, Cr and K levels. The difference between groups 2 and 3 was not significant in these parameters, except that group 3 had a lower prevalence of DM and higher baseline prealbumin level. There were no significant differences in the PD vintage, residual renal function, peritoneal Kt/V, BMI and MAP among the 3 groups (Table 1). By simple linear regression, we showed that APR positively correlated to age, hs-CRP level and DM, but negatively correlated to nPCR. In the multiple linear regression model 1, we showed that APR was positively associated with hs-CRP and negatively associated with nPCR (Table 2). After further including DM into the multiple linear regression analysis (model 2), we found that APR was negatively correlated to nPCR but positively correlated to DM. Moreover, we showed group 1 was associated with higher prevalence of DM (OR: 2.40, 95% CI = 1.08–5.32, p = 0.032), lower nPCR (OR:0.19, 95% CI = 0.04–0.92, p = 0.039), and higher hs-CRP (OR: 1.04, 95%CI = 1.01–1.07, p = 0.024) after adjusting for age, sex, residual renal function, and MAP.

### 3. Hyperprealbuminemia negatively correlated to age and comorbidity

The characteristics of individuals with hyperprealbuminemia was presented in Table 3. Compared to patients with normoprealbuminemia, those with prealbumin higher than 40mg/dL were younger and with less co-morbid conditions, including DM. They also had lower hs-CRP, APR, death rate, and higher serum albumin level.

### 4. High serum albumin to prealbumin ratio (APR) and normal prealbumin predict higher long-term mortality risk

**Fig 1:**
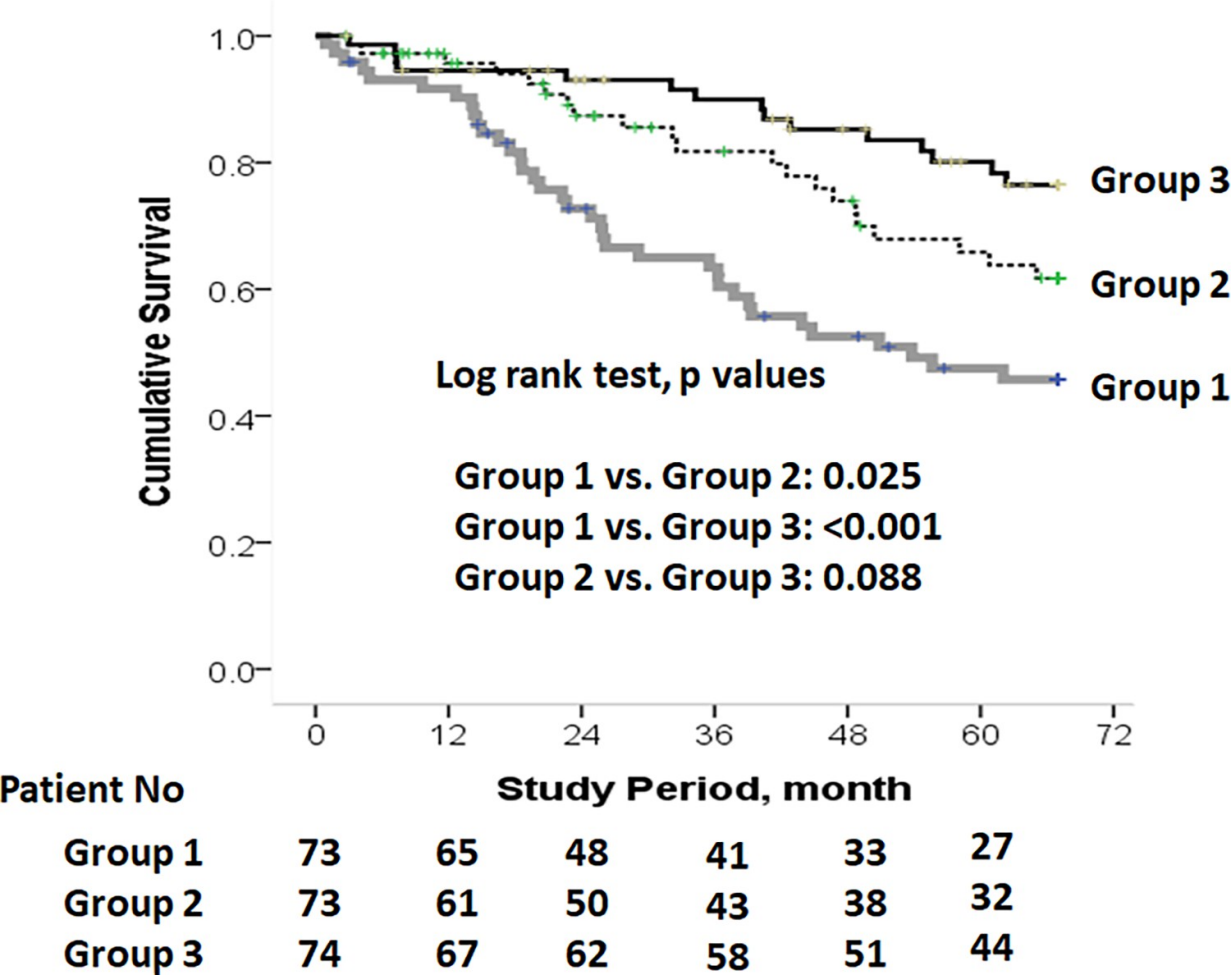
Kaplan-Meier survival analysis of cumulative all-cause mortality among three groups of patients based on tertile of albumin to prealbumin ratio. After 5 years’ follow-up, group 1 had lower cumulative survival rate than the other two groups (i.e., groups 2 and 3). There was no survival difference between groups 2 and 3.

Our results showed that group 1 had a lower cumulative survival than the other two groups after a median follow-up of 55.2 months (*p* values: groups 1 vs. 2 = 0.025; groups 1 vs. 3 <0.001). There was no significant difference in mortality between group 2 and 3 (p = 0.088) (Fig 1). After adjusted for age at the start of study, sex, and PD vintage, we showed that group 1 was significantly associated with higher mortality risk relative to group 2 and 3 (model 1 in Table 4). After further adjusted for prevalent DM, group 1 still had a higher mortality risk compared with group 3, but the difference between group 1 and 2 attenuated (model 2 in Table 4).

In Fig 2, we showed that patients in group 3 and with hyperprealbuminemia had significantly lower mortality risk as opposed to those in group 1 and with normoprealbuminemia, but patients in group 2 and with hyperprealbuminemia did not show a significant difference probably because of small sample size (n = 17). Additionally, we observed that the survival benefit of patients in groups 2 or 3 and with normoprealbuminemia attenuated.

By univariable Cox regression, we showed that higher APR, older age, DM as opposed to non-DM, higher Davies score (with extraction of DM), and prealbumin level lower than 40mg/dL were associated with higher risk of long-term mortality among PD patients. After adjusting for variables in Table 5, our results showed that higher APR is an independent predictor of long-term mortality, with 2% higher in mortality risk for every 1-unit increment of APR (p<0.001).

**Table 5:** Predictors of mortality by Cox proportional hazard model.

	Univariate analysis				Multivariate analysis			
		95% C.I. for HR				95% C.I. for HR		
	HR	Lower	Upper	p-values	HR	Lower	Upper	p-values
Albumin to Prealbumin ratio	1.03	1.02	1.04	<0.001	1.02	1.01	1.03	<0.001
Age at start of study, year	1.06	1.04	1.08	<0.001	1.05	1.03	1.08	<0.001
DM, y or n	2.93	1.84	4.66	<0.001	2.29	1.37	3.81	0.001
Davies score—DM	1.75	1.36	2.24	<0.001	1.46	1.12	1.90	0.005
mean arterial pressure, mmHg	0.99	0.97	1.00	0.04	1.00	0.99	1.02	0.787
PD vintage, months	1.00	1.00	1.01	0.62	1.00	1.00	1.01	0.220
Pre-albumin > 40mg/dL, y or n	0.41	0.23	0.73	0.003				
nPCR, g/kg/day	0.34	0.10	1.15	0.08				
hs-CPR, mg/dL	1.01	0.99	1.02	0.30				

Abbreviations: DM, diabetes mellitus, PD: peritoneal dialysis, nPCR: Normalized protein catabolism rate, hs-CRP: High sensitivity C-reactive protein.

### 5. Area under curve (AUC) of prealbumin is larger than that of albumin

**Fig 3:**
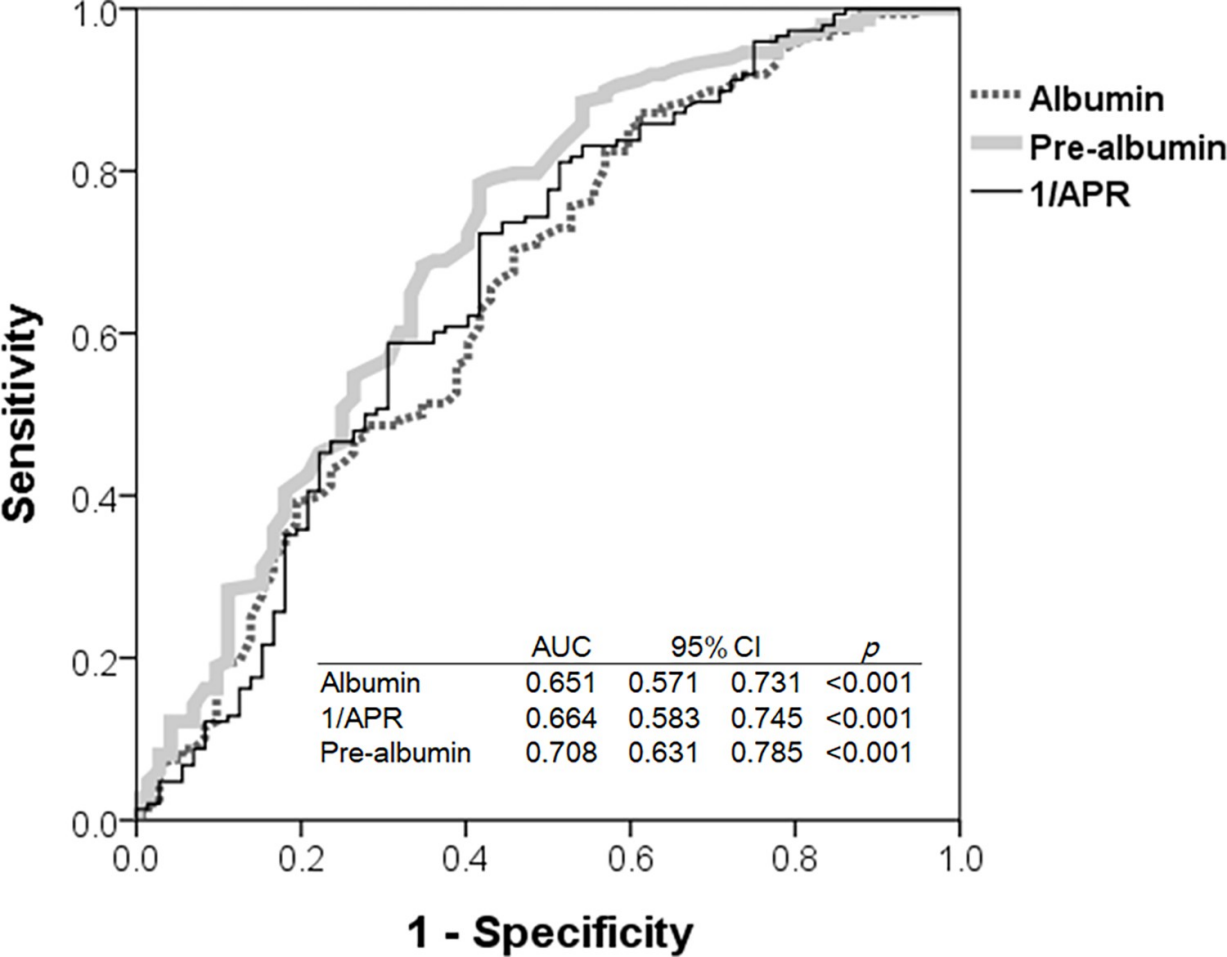
Receiver operating characteristic (ROC) curves of prealbumin, albumin and reciprocal of APR (1/APR). AUC value (0.708) of prealbumin was larger than either 1/APR (0.664) or albumin (0.651). The optimal cut-off of ROC in prealbumin was 33.5 mg/dL.

By using ROC curve for predicting survival probability (Fig 3), we observed that AUC value (0.708) of prealbumin was larger than either 1/APR (0.664) or albumin (0.651). The optimal cut-off value of ROC in prealbumin was 33.5 mg/dL.

## Discussion

In this study, we observed that PD patients with higher APR were characterized by lower nPCR, higher hs-CRP, and more co-morbidities, suggesting that high APR could be an indicator of malnutrition, inflammation, and protein catabolism. We also demonstrated that patients with high APR, i.e., group 1, had a higher mortality risk than those with middle or low APR. The mortality risk was 2% greater for every 1-unit increment of APR.

Normoalbuminemia (≥ 3.5 g/dL) has been considered a positive prognostic factor among dialysis patient [13]. However, among individuals in the high APR group (group 1), nearly 60% had normoalbuminemia but more than 90% had normoprealbuminemia. This discrepancy suggested that the combination of albumin and prealbumin as APR may be an alternative predictor of long-term clinical outcomes.

We noticed a general upward shift in prealbumin levels in PD patients, with almost all (96.8%) of the study population having normal or supernormal serum prealbumin levels. This logically amplifies the denominator and lowers the value of APR. Thus, the degree of hyperprealbuminemia seems to be inversely proportional to the APR level. Hyperprealbuminemia accounts for 0%, 23.3% and 82.4% in groups 1, 2, and 3, respectively, leading to highest APR in group 1 and lowest in group 3. Group 1 is characterized by slightly hypoalbuminemia and “normal” prealbumin level based on “reference range”, but in essence relatively lower than the other two counterparts, in which the degree of difference in prealbumin (denominator) exceeding that of albumin (nominator), thus magnifying the ratio. On the other hand, prealbumin levels in groups 2 and 3 were 1.33 and 1.66 times higher than in group 1, respectively, but albumin levels were only 1.04 and 1.05 times higher, leaded to lower level of APR in these two groups. In this regard, the APR is attributed far more to prealbumin than albumin levels.

The mean and median levels of prealbumin in the total study population are 36.9mg/dL and 37.0mg/dL, respectively. It implies that some patients with prealbumin level of 20-40mg/dL may be in a sub-optimal or even sub-normal state, whereas hyperprealbuminemia is actually optimal. Individuals with hyperprealbuminemia also showed better nutrition and fewer co-morbidities, than those with normoprealbuminemia, resulting in better outcomes in groups 2 and 3.

Although the best cutoff value for our data in ROC of prealbumin is 33.5mg/dL, it remains uncertain what the optimal range is for PD patients. Based on the recommendation by the consensus of the International Society of Renal Nutrition and Metabolism, the goal of serum prealbumin should be maintained higher than 30mg/dL in chronic kidney disease patients [14]. In our study population, 21% had serum prealbumin lower than 30 mg/dL, with 96% in group 1, and only 2% in groups 2 and 3 each having prealbumin level lower than 30 mg/dL. It implied that group 1 was under a more profound energy and protein depletion status than the other two groups.

In addition to enhanced hepatic synthesis in response to peritoneal albumin loss [10], another presumed mechanism of elevation in prealbumin level in chronic renal failure is the impairment in degradation by renal tubules [15]. However, our data showed that individuals in group 3 had high proportion of daily urine output of > 750 ml, despite renal CCr showed no difference. Future research is needed to investigate the underlying mechanism of elevation in prealbumin level among PD patients. Regardless, our results support that prealbumin has a critical role in APR and is an independent prognostic indicator among patients receiving PD [7].

Prealbumin has a shorter half-life of 48 hours and is a very sensitive indicator to protein catabolism [5]. It responds more rapidly to protein intake and anabolism than albumin, so it is a real-time marker rather than the state of several weeks ago reflected by albumin. We speculate that shorter half-life makes prealbumin more susceptible to inflammation stress when compared with albumin. Higher inflammation stress in group 1 resulted in a more prominent breakdown and impaired synthesis of prealbumin than albumin, which widens the gap between the levels of these two biomarkers and presents as the high APR.

The reference range of prealbumin applied in routine practice could be confusing when interpreting the status of PD patients, as 96.8% of patients were normal or even higher in prealbumin level, a unique feature of generally elevated prealbumin level in PD patients. Therefore, combination of albumin and prealbumin, seems to be a better strategy to interpretate the nutrition and inflammation status of PD patients.

Group 1 was characterized by lower serum albumin, prealbumin, Cr, K, and BUN, lower nPCR and higher hs-CRP levels, implying that high APR is associated with malnutrition and subclinical inflammation [16,17]. Lower BUN and nPCR over time have been linked to inadequate protein and energy intake and increased risk of mortality in HD patients [16]. In addition, low serum Cr and K levels were reported to correlate with less lean muscle mass and high catabolic rate in dialysis patients [18–21]. Our study showed that group 1 had higher prevalence of DM and co-comorbidities, indicating that they are under more inflammation stress, leading to protein degradation. Combining inadequate protein intake, malnutrition, and excessive catabolism, it eventually resulted in high mortality risk in group 1. Therefore, high APR could be a surrogate of malnutrition-inflammation-atherosclerosis (MIA) syndrome and a prognostic indicator in ESRD patients receiving long-term PD. High APR presenting with protein malnutrition and lean body mass reduction is a typical presentation of protein energy wasting (PEW) [22], since it negatively correlated to serum Cr, K, albumin, prealbumin, and cholesterol levels. We hypothesize that high APR, especially with a value higher than 100, i.e., mean value, is a good indicator to reflect the severity of PEW in PD patients.

There are several strengths in this study. First, we demonstrated the prognostic predictive value of APRs, which is a combination of the predictive power of serum albumin and prealbumin over 5-year observation among PD patients. Second, we pointed out that normoprealbuminemia should not be regarded as “normal” in PD patients, since normoprealbuminemia accounts for more than 90% in group 1 who have high long-term mortality risk. Last, we highlighted hyperprealbuminemia is a favorable outcome predictor in PD patients compared to normoprealbuminemia. However, our study bears some limitations. While subjective global assessment (SGA) and anthropometric measurements such as mid-arm muscle circumference, skin fold thickness and hand-grip strength has been recognized as more reliable methods for PEW evaluation in CKD patients [23,24], these data are not available in this study. However, based on our biochemical data, we suppose patients with high APR may have low SGA score and less muscle strength. Lastly, while we found APR in PD patients is about 77% of that in HD counterparts in our unpublished data, future studies are needed to examine whether our conclusion is generalizable to HD patients.

In conclusion: High APR is associated with low nPCR, BUN, Cr, K, and elevated hs-CRP, as well as more co-morbid conditions, suggesting that it represents malnutrition superimposed with persistent inflammation, even with normal level of prealbumin. These facts link high APR, especially more than 100, to high mortality risk in PD patients. On the other hand, hyperprealbuminemia, contributing to APR lowering, is a favorable prognostic predictor. Due to generally elevated prealbumin level in PD patients, clinicians may misinterpret these patients’ nutritional and inflammatory status when relying solely on the reference range of serum prealbumin level. Instead, APR by combining albumin and prealbumin is a surrogate of PEW syndrome and has a good predictive value in long-term outcomes among PD patients.

## Supporting information

S1 Dataset (XLSX)
